# Encapsulation of Thymol in Gelatin Methacryloyl (GelMa)-Based Nanoniosome Enables Enhanced Antibiofilm Activity and Wound Healing

**DOI:** 10.3390/pharmaceutics15061699

**Published:** 2023-06-09

**Authors:** Maryam Moghtaderi, Saba Bazzazan, Ghazal Sorourian, Maral Sorourian, Yasaman Akhavanzanjani, Hassan Noorbazargan, Qun Ren

**Affiliations:** 1 School of Chemical Engineering, College of Engineering, University of Tehran, Tehran 1417935840, Iran; 2 Department of Community Medicine, Mashhad Branch, Islamic Azad University, Mashhad 1477893855, Iran; 3 Department of Molecular and Cellular Biology, Faculty of Advance Sciences and Technology, Tehran Medical Sciences, Islamic Azad University, Tehran 1477893855, Iran; 4 Department of Biotechnology, School of Advanced Technologies in Medicine, Shahid Beheshti University of Medical Sciences, Tehran 1517964311, Iran; 5 Laboratory for Biointerfaces, Empa, Swiss Federal Laboratories for Materials Science and Technology, 9014 St. Gallen, Switzerland

**Keywords:** wound healing, antibacterial, antibiofilm, cell migration, Thymol, niosome, gelatin methacryloyl (GelMa)

## Abstract

Non-healing wounds impose huge cost on patients, healthcare, and society, which are further fortified by biofilm formation and antimicrobial resistance (AMR) problems. Here, Thymol, an herbal antimicrobial agent, is utilized to combat AMR. For efficient delivery of Thymol gelatin methacryloyl (GelMa), a hydrophilic polymeric hydrogel with excellent biocompatibility combined with niosome was used to encapsulate Thymol. After optimization of the niosomal Thymol (Nio–Thymol) in the company of GelMa (Nio–Thymol@GelMa) to achieve maximum entrapment efficiency, minimum size, and low polydispersity index, the Thymol release peaked at 60% and 42% from Nio–Thymol@GelMa in medium with pH values of 6.5 and 7.4 after 72 h, respectively. Furthermore, Nio–Thymol@GelMa demonstrated higher antibacterial and anti-biofilm activity than Nio–Thymol and free Thymol against both Gram-negative and Gram-positive bacteria. Interestingly, compared with other obtained formulations, Nio–Thymol@GelMa also led to greater enhancement of migration of human dermal fibroblasts in vitro, and higher upregulation of the expression of certain growth factors such as FGF-1, and matrix metalloproteinases such as MMP-2 and MMP-13. These results suggest that Nio–Thymol@GelMa can represent a potential drug preparation for Thymol to enhance the wound healing process and antibacterial efficacy.

## 1. Introduction

Wound infections are characterized by the occurrence of erythema, pain, tenderness, heat, and edema, as well as the pathogenicity and aggressiveness of the pathogens. They can lead to severe conditions and slow down the wound rehabilitation process [1]. Gram-negative *Pseudomonas aeruginosa*, *Escherichia coli*, *Klebsiella pneumoniae*, and Gram-positive *Staphylococcus aureus* bacteria are among the most significant causes of wound infections, partially due to their outstanding antibiotic resistance [2]. To overcome these antibiotic-resistant pathogens, alternative strategies are being developed [3]. Furthermore, the development of putative biofilms often occurs in chronic wounds [4], which are challenging to eliminate and can only partially react to drugs [5,6].

Many natural compounds that originated from plants have been reported to possess antibacterial, anti-biofilm, and wound healing properties. One of such known compounds is Thymol, which can be extricated from several plants such as Thymus, Satureja, Euphrasia rostkoviana, Lippia, and Coridithymus [7]. In the past, essential oils that contain Thymol have been applied for their doable assistance in medical treatment against inflammation and fungal and bacterial infections. Especially in the last two decades, many in vitro studies have been performed to approve the antimicrobial features of Thymol [8]. Since many antibiotic agents lose efficacy after a certain time of treatment, a combination of nature-based compounds such as Thymol can be beneficial [9]. Further, Thymol can downregulate virulence and antibiotic resistance genes when combined with antibiotics [9]. Thymol (2-isopropyl-5-methylphenol) is a monoterpene compound with proven effects on a variety of pathogenic bacteria, including *S. aureus*, *E. coli*, *Listeria monocytogenes*, and *Shigella sonnei* [10]. Some of the mechanisms by which Thymol suppresses bacterial growth are associated with its disruptive effects on the cell membrane of pathogens, reduction of bacterial biofilm and motility, inhibition of membrane-bound ATPases, and suppression of efflux pumps [10,11]. Due to the poor bioavailability of Thymol, drug delivery systems have been utilized for improving the control of drug release, as well as avoiding drug imbalances in the vascular system [12,13].

Niosomes are one of the most potent nano-vehicles and can be an outstanding option for controlled drug release since they are biocompatible, nontoxic, and capable of delivering substantial quantities of the targets drugs [12,14,15]. It has been reported that noisome-containing tannic acid can diminish biofilm-forming capability and down-regulated biofilm gene expression in *E. coli*, *K. pneumonia*, *P. aeruginosa*, and *S. aureus* strains [16,17]. In a very recent study, niosomes coated with chitosan were used to to deliver tetracycline antibiotics for wound healing, which showed negligible cytotoxicity and high efficacy against both Gram-negative and Gram-positive bacteria [18].

Hydrogels are cross-linkable polymers with a high water absorption capacity, and can imitate the characteristics of the natural extracellular matrix. Combined with nanomaterials, hydrogels can support the controlled release of active chemicals into the tissues [19]. Gelatin methacryloyl (GelMa) hydrogels are non-immunogenic materials with excellent biocompatibility, biodegradability, and moldability [20,21]. Owning to their distinctive properties and simple preparation, GelMA hydrogels offer promising potential in a broad range of biomedical applications, including drug delivery.

To the best of our knowledge, the combination of hydrogel with niosomes for Thymol delivery has not been reported so far. Previously, nanoparticles (lipid-based and polymer-based) were fabricated to delivery Thymol [22,23,24]. It was shown that Thymol-loaded polylactic acid (PLA) nanoparticles presented higher stability at various pHs, and their antimicrobial activity against *E. coli* was improved compared with that of free Thymol [24]. Encapsulating Thymol in liposome nanoparticles led to a more controlled and slower release compared with the free drug, and reduced bacterial attachment and prevented biofilm formation [23]. The current study aims to develop a niosome system with Thymol (Nio–Thymol) incorporated into GelMa (Nio–Thymol@GelMa) to improve its antibacterial, anti-biofilm, and wound healing activities. The obtained niosomes were investigated for their entrapment efficiency and drug release kinetics. Characterizations of these vehicles were analyzed by applying DLS (dynamic light scattering method), TEM (transmission electron microscope), and SEM (Scanning Electron Microscope). The release profile and stability were investigated as well. The antibacterial and anti-biofilm properties were evaluated against both Gram-negative and Gram-positive bacteria. Cytotoxicity was assessed with human foreskin fibroblast. A scratch assay was performed to study the impact of the fabricated niosomes on cell migration.

## 2. Materials and Methods

### 2.1. Materials

Thymol, Span^®^ 60 (sorbitan monostearate), and cholesterol were purchased from Millipore Sigma (Burlington, MA, USA) and used without further purification. Chloroform, methanol, dimethyl sulfoxide (DMSO), dialysis membrane (MWCO 12,000 Da), sodium dodecyl sulphate (SDS), phosphate-buffered saline (PBS), and Amicon (Ultra-15-Membrane, MWCO 30,000 Da) were acquired from Merck Chemical Co. (Darmstadt, Germany). The HFF (Human Foreskin Fibroblast) cell lines were purchased from Pasteur Institute Cell Bank (Tehran, Iran). Medium RPMI-1640 (Dulbecco’s Adjusted Eagle Medium), formaldehyde, trypsin-EDTA, Trypan blue, fetal bovine serum (FBS), phosphate-buffer, 3-(4,5-dimethylthiazol-2-yl)-2,5-diphenyltetrazolium bromide (MTT), and penicillin/streptomycin (PS) 100 X were acquired from Gibco, ThermoFisher Scientific (Waltham, MA, USA). The RNA Extraction (Cat No. ER101-01) and cDNA Synthesis kits (Cat No. AE301-02) were purchased from Transgene Biotech Ltd. (Beijing, China). *S. aureus* (ATCC 6538), *K. pneumonia* (ATCC 13883), *Bacillus subtilis* (*B. subtilis*) (ATCC 21332), and *P. aeruginosa* (ATCC 15442) were obtained from Pasteur Institute of Iran. Mueller Hinton Broth, Mueller Hinton Agar, Barium chloride, and H_2_SO_4_ were bought from Merck Co. (Darmstadt, Germany). Crystal violet and methanol were purchased from Millipore Sigma (Burlington, MA, USA) and used without further purification. Tryptic soy broth was purchased from ThermoFisher Scientific (Waltham, MA, USA).

### 2.2. Optimization of Niosomal Formulations

Response surface methodology (RSM) was used to optimize niosomal formulations. To investigate the relationship between a set of independent variables and the dependent variables by fitting the data using a polynomial equation, three numerical parameters (Span 60 content (mM), Cholesterol content (mM), and Volume hydration (mL)) were selected to study their impact on niosomal particle size (nm), polydispersity index (PDI), and entrapment efficacy (EE). The polynomial equation was obtained using Design-Expert software (Version 10.0.10, Stat-Ease, Inc., Minneapolis, MN, USA). The optimum formulation was selected as the one possessing the minimum size and PDI of the niosomes and the maximum EE. The best-fitted model for the statistical analysis was considered significant when *p*-value < 0.05 and the equations of the most accurate model were achieved. The desirability index was used in the optimization of the Box–Behnken method [25]. Using the point prediction method, the optimal formulation was chosen for further study. Table S1 represents these factors and their levels.

### 2.3. Synthesis of Niosomal Formulations

**Figure 1:**
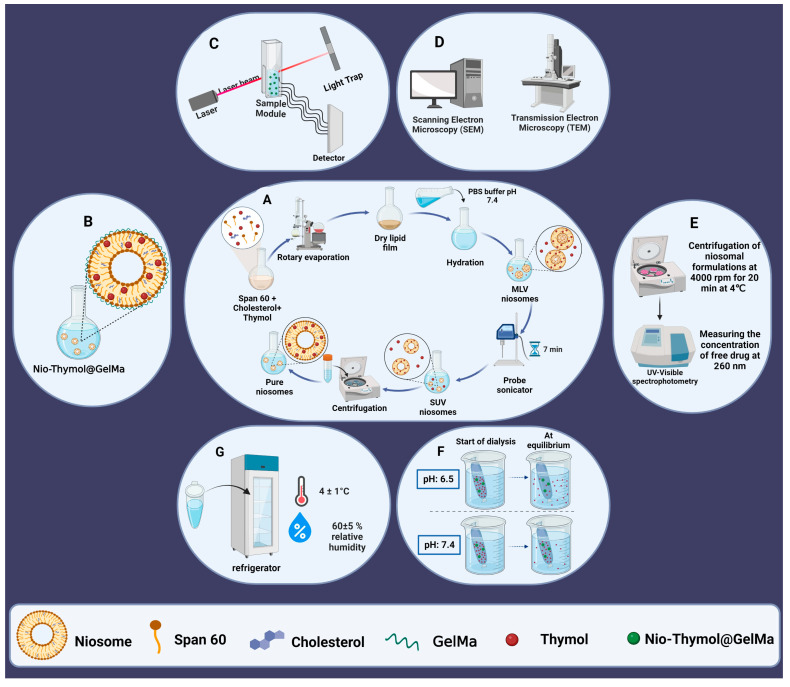
Fabrication and characterization of the niosomal formulations. (**A**) Preparation of niosomes using thin-layer hydration method, MLV: multilamellar vesicles, SUV: small uni-lamellar vesicles; (**B**) functionalizing niosomes with GelMa; (**C**) size determination; (**D**) morphology study; (**E**) entrapment efficacy study; (**F**) in vitro drug release study; and (**G**) stability study.

Niosome-loaded Thymols (Nio–Thymol) were prepared using the thin-film hydration method [26]. Briefly, Thymol (10 mg), cholesterol, and Span^®^ 60 were dissolved in 9 mL of chloroform/methanol (2:1; *v*/*v*), and the organic solvent was then evaporated under vacuum using a rotary evaporator (Heidolph Instruments, Schwabach, Germany) at 60 °C and 150 rpm for 30 min until a thin dried film was formed in the bottom of the flask. The thin film was rehydrated at 60 °C and 150 rpm for 30 min using PBS (1×, 10 mL, pH 7.4). Subsequently, the niosomal dispersion was sonicated using a probe sonicator (UP50H compact laboratory homogenizer, Hielscher Ultrasonics, Teltow, Germany) for 7 min to homogenize size distribution (Figure 1A). The different niosomal formulations are listed in Table 1.

### 2.4. Surface Functionalization of the Optimized Niosomal Formulation

GelMa dispersion (0.7%, *w*/*v*) was prepared by sprinkling over boric acid/borax buffer (pH 7.4) at 80 °C while stirring. The mixture was stirred continuously until a clear dispersion was obtained. After cooling, GelMa dispersion was mixed with the optimized Thymol (1 mg/mL)-loaded niosomes at 80 °C while stirring for 1 h (Figure 1B). The obtained Nio–Thymol@GelMa was kept for 12 h at 4 °C to allow the removal of any entrapped air bubbles within the gel [27,28].

### 2.5. Physicochemical Characterization of the Synthesized Niosomes

#### 2.5.1. Size, Charge Surface, and Polydispersity Index (PDI)

The dynamic particle diameter and size distribution were determined with the dynamic light scattering method (DLS), using a computerized inspection system (Zetasizer, HAS 3000; Malvern Instruments, Malvern, UK) at 25 °C and 633 nm wavelength (Figure 1C).

#### 2.5.2. Morphology

Morphology of niosome vesicles was visualized using a transmission electron microscope (TEM, Zeiss EM900 Transmission Electron Microscope, Germany, accelerating voltage of 80 kV) and a Scanning Electron Microscope (SEM, NOVA NANOSEM 450 FEI model). The sample for TEM observation was prepared by placing a few drops of optimized niosome dispersion onto a 300-mesh carbon-formvar grid and allowing two minutes for absorption. The extra liquid was removed with a filter paper. The grade was washed and stained with ten drops of 2% uranyl acetate. For imaging with SEM, samples were dropped and dried on the SEM holder and coated with a layer of 100 Å gold for 3 min under argon at a pressure of 0.2 atm (Figure 1D).

#### 2.5.3. Entrapment Efficacy

The niosomal formulations were ultra-filtered (Eppendorf^®^ 580R centrifuge, Hamburg, Germany) with an Amicon Ultra-15-membrane at 4000 rpm for 20 min at 4 °C. The non-entrapped Thymol was separated from entrapped ones to determine the percentage entrapment efficacy (EE%) (Figure 1E). The free drug concentration was measured at 260 nm using a UV-Visible light spectrophotometer (UV-1700 PharmaSpec, Shimadzu, Kyoto, Japan). The following equation was used to calculate EE% [29].

EE%= [(A−B)/A]×100

where (*A*) is the initial Thy concentration for niosomal preparation and (*B*) is the concentration of non-entrapped Thy after centrifugation.

#### 2.5.4. In Vitro Drug Release and Kinetic Study

For evaluation of drug release, 2 mL of each sample was placed in a semipermeable acetate cellulose dialysis bag (MWCO 12 kDa), which was immersed in 50 mL of PBS-SDS (0.5% *w*/*v*; release medium). The assembly was agitated at 50 rpm using a magnetic stirrer in various pH conditions (7.4 and 6.5) at 37 °C for 72 h. At a specific time (1, 2, 4, 8, 24, 48, and 72 h), 1 mL of the release medium was withdrawn and replenished with the same volume of fresh PBS-SDS [30]. The amount of released drug at pre-determined intervals was measured at 260 nm using an ultraviolet light spectrophotometer (Figure 1F). The test was also done for free drug as control, in which the drug concentration was equivalent inside and outside the dialysis bag.

Different mathematical models were used to evaluate the release kinetics of Thymol from the samples: the Korsmeyer–Peppas model, the Higuchi model, the first-order model, and zero-order model [31,32,33,34]. The correlation coefficient (r) values obtained via regression of the plots derived from the above models were used to calculate the linear curve.

#### 2.5.5. Stability

The Nio–Thymol and Nio–Thymol@GelMa samples were stored at 25 ± 1 °C (room temperature) or 4 ± 1 °C (refrigeration temperature), at 60 ± 5% relative humidity for two months. The samples were analyzed for particle size, PDI, and percentage of drug remaining at certain time intervals (0, 30, and 60 days) for evaluation of the stability of the formulations (Figure 1G).

### 2.6. Antibacterial Tests

#### 2.6.1. Minimum Inhibitory Concentration (MIC)

The MICs of Thymol, Nio–Thymol, and Nio–Thymol@GelMa were examined using the micro-dilution method following CLSI (Clinical and Laboratory Standards Institute) guidelines and were performed in triplicate. *S. aureus* (ATCC 6538), *K. pneumonia* (ATCC 13883), *B. subtilis* (ATCC 21332), *and P. aeruginosa* (ATCC 15442) were used. A total of 200 μL of each different concentration (1.95 to 500 μg/mL) of Thymol, Nio–Thymol, and Nio–Thymol@GelMa was added to a 96-well plate, then 80 μL Müller–Hinton Broth (MHB) and 20 μL microbial suspension were added at the concentration of 1.5 × 10^8^ CFU/mL, followed by 24 h of incubation at 37 °C to determine the MIC values. MIC is the lowest concentration of the drug capable of inhibiting bacterial growth. In this test, negative and positive controls were drug-free wells with and without bacteria.

#### 2.6.2. Agar Diffusion Method

The antibacterial activity of Thymol, Nio–Thymol, and Nio–Thymol@GelMa against *S. aureus, K. pneumonia, B. subtilis,* and *P. aeruginosa* was carried out using the agar-well diffusion method. Briefly, strains were cultured on Müller–Hinton agar (MHA) plates at a density of 5 × 10^5^ CFU/mL. Wells (diameter of 6 mm) were cut in the agar and filled with 10 µL of sample solutions. The plates were incubated at 37 °C for 24 h. After incubation, the diameter of growth inhibition was measured using a ruler with up to 1 mm resolution.

#### 2.6.3. Time-Kill Assay

The time-kill study was determined using the 96-well plate technique [35]. In a typical procedure, Thymol, Nio–Thymol, and Nio–Thymol@GelMa were diluted up to concentrations equal to the MIC values. Then, 100 µL of test samples was added to each microtiter plate well, which was pre-loaded with 100 µL of each bacterial suspension containing 10^5^ CFU/mL and incubated at 37 °C. The optical density (OD 600 nm) was measured at 2, 4, 6, 24, and 48 h intervals using a microplate reader (EPOCH, Japan). The growth curve of bacteria was used as a positive control.

### 2.7. Anti-Biofilm Activity

A 96-well plate based on Crystal violet (CV) staining [36] was used to evaluate the anti-biofilm efficacy of Thymol, Nio–Thymol, and Nio–Thymol@GelMa. The biofilm-forming *S. aureus*, *B. subtilis*, *K. pneumonia*, and *P. aeruginosa* strains were cultured into 96-well microtiter plates for 24 h at 37 °C. The strains were then treated with MIC values of each sample for 24 h at 37 °C. Afterward, all the wells were washed with PBS three times and fixed with methanol for 15 minutes. The plate was air-dried for 30 minutes, and 0.1% CV solution was added to each well and incubated at room temperature for 20 min. After washing with distilled water, 33% acetic acid was added to each well, and absorbance was taken at 570 nm. The mean absorbance values of each sample were calculated and compared with the mean values of controls.

### 2.8. Cytotoxicity Assay

#### 2.8.1. Culture of HFF Cell Lines

Human Foreskin Fibroblast (HFF) cell lines were cultured at 37 °C in atmospheric conditions that were supplemented with 5% CO_2_. The culture medium consisted of RPMI-1640 fresh medium supplemented with 10% FBS and 1% penicillin/streptomycin (complete growth medium). The medium was aspirated after the cells reached 85–95% confluence. Detachment of the cell monolayer was performed using 0.25% (*w*/*v*) trypsin-EDTA. The detached cells were re-suspended in a complete growth medium, labeled trypan blue, and counted with a hemocytometer.

#### 2.8.2. Cell Viability Assay

Different concentrations (62.5, 31.25, 15.62, 7.81, 3.9, and 1.95 μg/mL) of Nio, Nio@GelMa, Thymol, Nio–Thymol, and Nio–Thymol@GelMa were added to the cultured HFF cells and incubated for 48 h. For evaluation of cell viability, the cells were incubated with 0.5 mg/mL of MTT for 4 h to reduce the colorless tetrazolium dye MTT to insoluble formazan, which has a purple color. The formazan was dissolved in 100 μL of DMSO for colorimetric determination of the oxidoreductase enzymatic activity. Absorbance was measured at 570 nm using a microplate reader (AccuReader, Metertech, Taiwan) and the cell survival rate was calculated using the formula: Percentage cell viability (%) = Optical Density_570_ treatment/Optical Density_570_ control × 100%. For control, HFF cells were incubated with RPMI medium without the test sample.

### 2.9. In Vitro Wound Scratch Assay

HFF (1 × 10^6^ cells) cells were seeded in 35 mm cell culture dishes in RPMI medium and incubated at 37 °C in humidified 5% CO_2_ for 24 h to attach. A wound was generated in the formed monolayers using 200 μL tips. Cells were washed twice with PBS, followed by treatment with media containing Nio, Thymol, Nio–Thymol, and Nio–Thymol@GelMa, respectively, at a MIC concentration, and incubation for 24 h. The induced scratch was photographed using a live-cell imaging system at 0 and 24 h. The area of open scratches in the captured photos was assessed using the Image Processing and Analysis Java (ImageJ) software (National Institutes of Health, Bethesda, MD, USA). The experiment was performed in triplicate, and data were analyzed using GraphPad Prism 8 software.

### 2.10. Quantitative Real-Time Polymerase Chain Reaction (qRT-PCR)

The HFF cells (1 × 10^8^ cells/well) were treated with Nio, Thymol, Nio–Thymol, and Nio–Thymol@GelMa for 48 h. The gene expression of the fibroblast growth factor-1 (FGF-1), matrix metallopeptidase 2 (MMP-2), and matrix metallopeptidase 13 (MMP-13) were assessed using real-time qPCR. Ice-cold RNX TM–PLUS solution was added to tubes containing the treated cells, and the mixture was vortexed, and incubated at room temperature for 5 min. Chloroform was then added to the tubes. After setting the cells on ice for 5 min, the samples were centrifuged at 12,000 rpm at 4 °C for 15 min. The supernatant were transferred to RNase-free 1.5 mL tubes that contained an equal volume of isopropanol, and centrifuged. The pellet of extracted RNA was re-suspended in 75% ethanol, re-centrifuged, and re-suspended in diethylpyrocarbonate (DEPC)-treated H_2_O. Complementary DNA (cDNA) was prepared from the extracted RNA by adding 10 μL of reaction buffer (2×), 5 μg of the extracted RNA, and 2 μL of Enzyme-Mix to enough DEPC-treated water in RNase-free tubes to make up 20 μL of solution. The mixture was incubated for 10 min at 25 °C and 60 min at 47 °C. The reaction was stopped by heating at 85 °C for 5 min, and the mixture was kept on ice until use. The primers for FGF-1, MMP-2, MMP-13, and the housekeeping gene ß-actin were designed using the National Center for Biotechnology Information database, and are listed in Table S2. The qRT-PCR was performed with the sense and antisense primers using an ABI 7000 Real-Time PCR system utilizing the SYBR Green MASTER Mix (Bio-RP). The 2^−ΔΔCT^ method was used to determine fold changes relative to the control group. Experiments were conducted in triplicate.

### 2.11. Statistical Analysis

Statistical analysis and curve fitting were performed using GraphPad Prism software version 8 (GraphPad Software, Inc., San Diego, CA, USA). Data from three independent experiments were expressed as means ± standard deviations. Statistical significance was determined with a one-way analysis of variance after validating the normality and homoscedasticity of the data sets. For all studies, statistical significance was pre-set at α = 0.05. Box–Behnken method was performed using Design-Expert software version 10 (Stat-Ease Inc., Minneapolis, MN, USA).

## 3. Results and Discussion

### 3.1. Optimization of Niosome Fabrication

**Figure 2:**
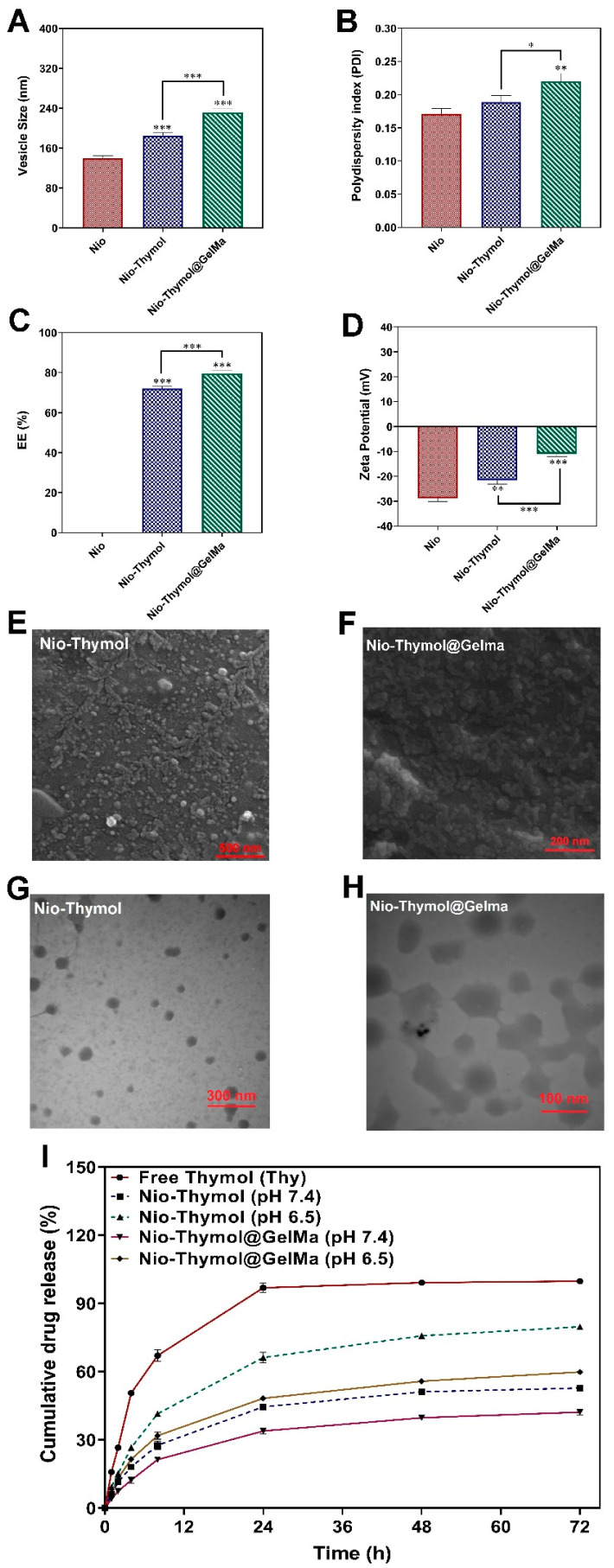
Comparison of size (**A**), polydispersity index (PDI) (**B**), entrapment efficiency (EE) (**C**), and Zeta potential (**D**) of Nio, Nio–Thymol, and Nio–Thymol@GelMa formulations. Data are the mean SD of three independent experiments. Significant difference specified as *** *p* < 0.001, ** *p* < 0.01, * *p* < 0.05. (**E**,**F**) SEM images of Nio–Thymol and Nio–Thymol@GelMa. (**G**,**H**) TEM images of Nio–Thymol and Nio–Thymol@GelMa. (**I**) In vitro drug release profile of Nio–Thymol and Nio–Thymol@GelMa in different pH.

Response surface methodology (RSM) was used to optimize niosomal formulations as detailed in SI. Three numerical parameters (Span 60 content, cholesterol content, and hydration volume) were selected to study their impact on niosomal particle size, polydispersity index (PDI), and entrapment efficacy (EE). The optimum formulation targeted the minimum size and PDI of the niosomes and the maximum EE. The analysis of variance, regression model, and accuracy and validity of models for responses are shown in Table S3, Table S4, and Table S5**,** respectively. Figure S1 shows the response surface plot of Nio–Thymol, the particle size, PDI, and EE (%). Finally, the optimum conditions for hydration volume, cholesterol, and Span 60 were projected to be 4.1 mL, 0.73 mM, and 8.31 mM, respectively (Table S6). All of the three response parameters were found to possess adequate desirability, namely about 0.88 for each (Table S6), verifying the suitability of the experimental design strategy. Under these optimized conditions, Niosomes, Nio–Thymol, and Nio–Thymol@GelMa with sizes of 139 ± 5, 184 ± 6, 231 ± 7 nm, PDI of 0.17 ± 0.00, 0.18 ± 0.01, 0.22 ± 0.01, respectively, and EE of 72 ± 1 and 79 ± 1% for Nio–Thymol and Nio–Thymol@GelMa, respectively, were fabricated (Figure 2A–C and Table S7).

### 3.2. Characterization of Nio–Thymol and Nio–Thymol@GelMa

#### 3.2.1. Morphology Study

The average size of the optimized nanoparticles measured by the DLS method were at about 139 nm, 184 nm, and 231 nm for Nio, Nio–Thymol, and Nio–Thymol@GelMa, respectively (Table S7). The obtained niosomes were further analyzed using SEM (Figure 2E,F) and TEM (Figure 2G,H). Nio–Thymol showed an approximate spherical shape and an almost smooth surface with a particle size of around 30–50 nm (Figure 2E), while Nio–Thymol@GelMa exhibited a scattered nearly spherical form with a distinct wall and aqueous core, and an average size of 70–100 nm (Figure 2F). The spherical forms of Nio–Thymol (Figure 2G) and Nio–Thymol@GelMa (Figure 2H) were confirmed via TEM images, showing similar sizes, as estimated with SEM. Moreover, the borders of the obtained niosomes can be clearly seen with TEM (Figure 2H). The polymerized hydrogel networks appeared to be responsible for the rise in the size of the Nio–Thymol@GelMa formulation [37]. The particle size dispersion was greater measured by DLS than that by SEM and TEM, which may be caused by the formation of the hydrodynamic shell in the aqueous condition for DLS measurement, likely dependent on niosome composition, roughness, unique shape, and interactions [38].

#### 3.2.2. Surface Charge Analysis

Zeta potential of the optimum preparations was analyzed using a zeta sizer, and Niosome, Nio–Thymol, and Nio–Thymol@GelMa exhibited values at −28 ± 1, −21 ± 1, and −10 ± 1, respectively (Figure 2D, Table S7). The results showed that encapsulating Thymol in niosomes leads to an increase in the zeta potential of the niosome, which is likely caused by the multi-component nature of Thymol (mainly monoterpene) [39]. In addition, adding GelMa to the formulation revealed even higher zeta potential due to the incorporation of relatively more positively charged GelMa into the niosomal membranes.

#### 3.2.3. In Vitro Release Study

Thymol release from the formulated Nio–Thymol and Nio–Thymol@GelMa was tested in neutral (pH 7.4) and acidified (pH 6.5) PBS solutions, and the release of Thymol was recorded for up to 72 h. Drug encapsulation in niosomes clearly reduced Thymol release rate (Figure 2I) and the addition of GelMa further enhanced this reduction. After 24 h at pH 7.4, Nio–Thymol and Nio–Thymol@GelMa released about 44% and 34% Thymol, respectively, and at pH 6.5, about 66% and 48%, respectively (Figure 2I). After 24 h, the release rate slowed down dramatically for all samples. After 72 h, Thymol release peaked at 53% and 80% for Nio–Thymol and 42% and 60% for Nio–Thymol@GelMa at pH values of 7.4 and 6.5, respectively. The enhanced drug release at pH values of 6.5 was likely caused by the spurring of the hydrolysis process of surfactants in an acidic environment [40]. The reduced release rate with GelMA incorporation has been observed previously [41]. It was reported that the GelMA concentration has an evident impact on the drug release kinetics, likely due to the need for additional diffusion through the GelMA layer, thereby enhancing the release longevity. Table S8 demonstrates the different release kinetic models for Thymol, Nio–Thymol, and Nio–Thymol@GelMa.

#### 3.2.4. Stability Study

The stability of the optimized niosomes was examined by determining size, PDI, and EE after two months of storage in a refrigerator (4 ± 2 °C) and room temperature (25 ± 2 °C) (Figure S2). It was found that at both temperatures, the size and PDI of the niosomal formulations elevated with increasing storage time, with less increase at 4 °C than 25 °C, which was likely due to lower mobility of niosomes at lower temperatures [16,42]. After 2 months, the particle size increased from 184 to 377 nm at 25 °C and to 315 nm at 4 °C for Nio–Thymol, and from 231 to 356 nm at 25 °C and 327 nm at 4 °C for Nio–Thymol@GelMa. The EE changed as well during storage, with 4 °C leading to less reduction than 25 °C and with Nio–Thymol@GelMa showing less change than Nio–Thymol. After 2 months, the EE decreased from 72% to 59% at 25 °C and 64% at 4 °C for Nio–Thymol, and from 79% to 68% at 25 °C and 73% at 4 °C for Nio–Thymol@GelMa. The greater mobility of the vesicles at elevated temperatures can indeed induce substantial drug leakage at 25 °C [16]. The addition of methacryloyl side groups permitted the GelMA molecule to polymerize quickly, resulting in covalent bonding via the formation of a methacryloyl backbone [43] and thus reducing the leakage of drug. The reduced alternation in EE of Nio–Thymol@GelMa was also in agreement with the less changes in its particle size and PDI during storage (Figure S2).

### 3.3. Antibacterial Assay

#### 3.3.1. Minimum Inhibitory Concentration (MIC) and Agar Diffusion Method

**Figure 3:**
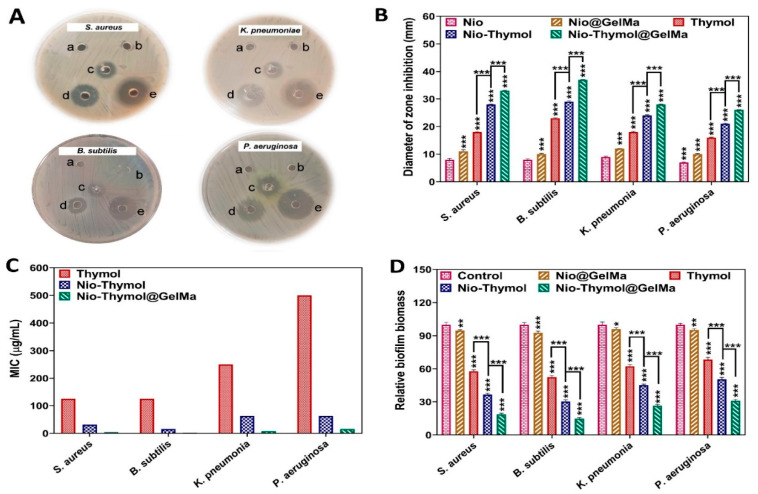
Antimicrobial activities of Thymol, Nio–Thymol, and Nio–Thymol@GelMa formulations against *S. aureus*, *B. subtilis*, *K. pneumoniae*, and *P. aeruginosa* standard strains. (**A**) Agar diffusion test with (a) Nio, (b) Nio@GelMa, (c) Thymol, (d) Nio–Thymol, (e) and Nio–Thymol@GelMa; (**B**) statistical data of the inhibition zones (*** *p*-value < 0.001); (**C**) MIC values; and (**D**) normalized antibiofilm activity after treatment, using control biofilms treated with PBS as 100% (* *p*-value < 0.05, ** *p*-value < 0.01, and *** *p*-value < 0.001).

The antimicrobial activity of the fabricated niosomes was analyzed first using the classical agar diffusion assay. It was found that Nio–Thymol@GelMa led to the largest inhibition zone for the tested strains (Figure 3A,B). MIC measurement was further measured to confirm the obtained results. The mean MIC values of Thymol, Nio–Thymol, and Nio–Thymol@GelMa were measured to be 125, 15.62–31.25, and 1.95–3.9 µg/mL against Gram-positive (*S. aureus* and *B. subtilis*) and 250–500, 62.5, and 7.81–15.62 µg/mL against Gram-negative strains (*K. pneumonia* and *P. aeruginosa*), respectively (Figure 3C). The MIC values of Nio–Thymol@GelMa against all tested strains were significantly lower in comparison with those of both Nio–Thymol and pure Thymol (*p* < 0.001). The observed increased antimicrobial activity of Nio–Thymol than free Thy might be attributed to the greater engagement with bacterial membranes and absorption of niosomes by microorganisms [44], which allowed better targeting of the drug to pathogens. Furthermore, the increased antibacterial activity of Nio–Thymol@GelMa formulation can be attributed to its more positively charged surface than Nio–Thymol (Figure 2D), which can permit better interaction with the negatively charged bacterial membrane, and consequently better targeting and deeper penetration of Thymol to bacterial cells [45,46].

#### 3.3.2. Time-Kill Assay

Time-kill profiles of Thymol, Nio–Thymol, and Nio–Thymol@GelMa formulations were studied against *S. aureus, B. subtilis, K. pneumonia*, and *P. aeruginosa*. Similar to the MIC assay results, encapsulation of Thymol into niosome boosted its antibacterial efficacy. Utilization of GelMA further increased the antibacterial performance of Thymol (Figure S3). During 48 h of incubation, a steady inhibition of bacterial growth was detected for Nio–Thymol@GelMa, while the free drug allowed significant bacterial growth. Such distinct antimicrobial activity profiles for free and encapsulated drugs have been reported previously [47]. Heidari et al. showed that the tannic acid-loaded niosomes inhibited bacterial growth slowly but consistently for 72 h. In contrast, the free drug was depleted in the first hours [47]. The growth of bacterial cells exposed to the antimicrobial drugs showed an increased lag phase, reduced growth rate, or/and lowered final biomass. In the case of the encapsulated drug, the drug was progressively discharged over time (Figure 2I), resulting in a relatively low and long antibacterial activity profile (Figure S3).

#### 3.3.3. Anti-Biofilm Activity

The antibiofilm activity of Nio@GelMa, Thymol, Nio–Thymol, and Nio–Thymol@GelMa formulation was assessed against *S. aureus*, *B. subtilis*, *K. pneumoniae*, and *P. aeruginosa* biofilms (Figure 3D). Biofilm analysis using CV staining revealed that Thymol, Nio–Thymol, and Nio–Thymol@GelMa remarkably decreased biofilms of both Gram-positive and Gram-negative strains, namely 57%, 36%, 18% for *S. aureus*; 52%, 30%, and 14% for *B. subtilis*; 62%, 45%, and 26% for *K. pneumoniae*; and 68%, 50%, and 30% for *P. aeruginosa*, respectively, compared with the control treated with PBS. There was no significant difference between the biofilms treated with Nio@GelMa and the control. These results demonstrated the high efficacy of Nio–Thymol@GelMa in treating biofilms.

### 3.4. Wound Healing

#### 3.4.1. Cytotoxicity

**Figure 4:**
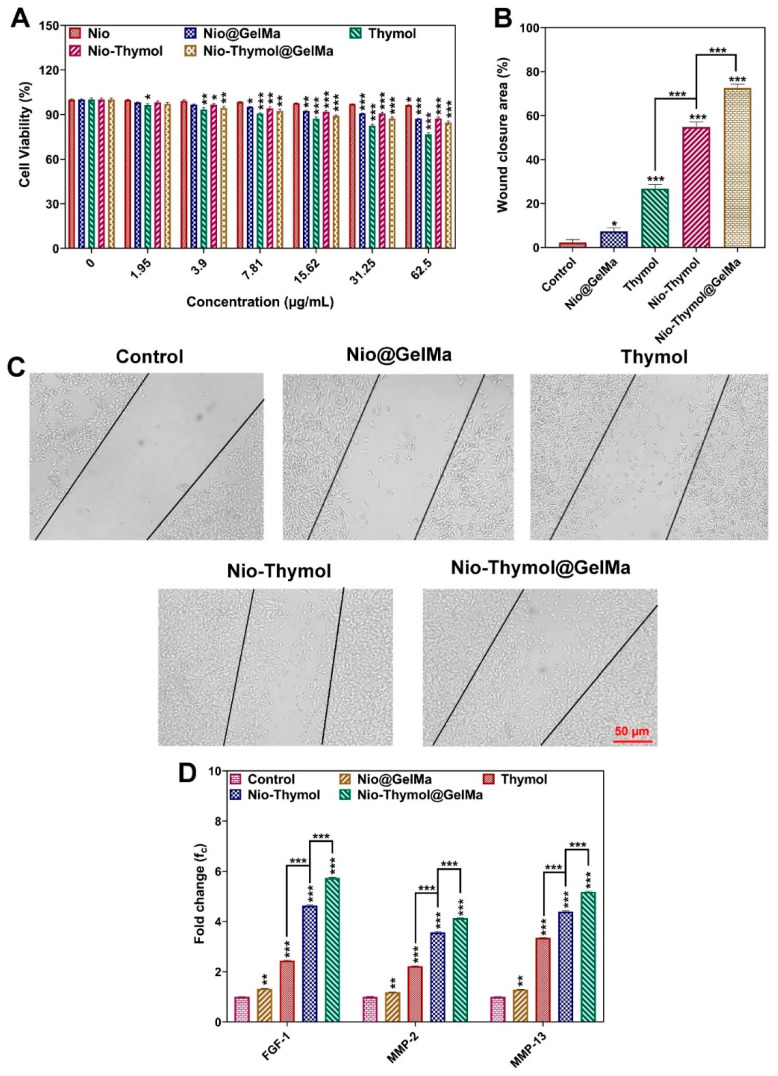
(**A**) HFF cell viability exposed to different concentrations of Nio, Nio@GelMa, Nio–Thymol@GelMa, Nio–Thymol, and Thymol. (**B**) Statistical analysis of cell migration in the in vitro wound (scratch) experiments for normal cells treated with control Nio, Nio@GelMa, Thymol, Nio–Thymol, and Nio–Thymol@GelMa formulations. (**C**) Images of wound scratch assay after 24 h. (**D**) Expression analysis of growth factors (FGF-1, MMP-2, and MMP-13) in samples treated with Nio@GelMa, Thymol, Nio–Thymol, and Nio–Thymol@GelMa formulations using qPCR (* *p*-value < 0.05, ** *p*-value < 0.01, and *** *p*-value < 0.001).

Cytotoxicity is one of the most essential considerations for employing drug formulations in medical applications. The cell cytotoxicity of the hereby prepared formulations (Nio, Nio@GelMa, Thymol, Nio–Thymol, and Nio–Thymol@GelMa) was tested against HFF cell lines after 48 h of treatment and compared with the control group treated with medium (Figure 4A). While niosomes (Nio) showed negligible cytotoxicity even at the highest concentration of 62.5 µg/mL (*p*-value < 0.05), other applied formulations slightly reduced cell viability at this concentration (*p*-value < 0.001); however, this still allowed viability for more than 75%. As expected, encapsulation of Thymol into the niosome and the addition of GelMa decreased its toxicity compared with that of the free Thymol. These results demonstrated that niosomes–GelMa could be comprehended as non-toxic and safe drug carriers. Particularly in the presence of GelMa, the controlled release of Thymol from the swollen or degrading hydrogels can reduce the potential toxicity to cells.

#### 3.4.2. In Vitro Cell Migration

In vitro wound scratch assays with HFF cells were performed to investigate the impact of Nio@GelMa, Thymol, Nio–Thymol, and Nio–Thymol@GelMa formulations on cell invasion and migration. The cell migration rate was tracked by the closing of the scratch (Figure 4B,C). All formulations were found to be able to promote normal cell migration (*p* < 0.001). However, cells treated with Nio–Thymol@GelMa showed significantly higher migration (73%) than those treated with Nio@GelMa (7%), Thymol (27%), and Nio–Thymol (55%), suggesting that Nio–Thymol@GelMa can induce the closing of the scratch area in HFF cells remarkably better than the other applied formulations (*p* < 0.001). These results also suggested that all formulations containing Thymol can promote cell migration, while Nio@GelMa has limited action. It has been reported previously that Thymol-loaded nanoparticles promoted cell regeneration [48]. Thymol is known to be an oxygenated composition from monoterpene that may act as a crucial modulator and affect fibroblast metabolism and collagen synthesis. These effects could be the reason for the usage of Thymol in chronic wound management [49].

#### 3.4.3. Expression of Growth Factors upon Thymol Treatment

The potential wound-healing effect of the prepared formulations was further investigated by analyzing the gene expression of growth factor FGF-1 and the matrix metalloproteinases MMP-2 and MMP13. FGF-1 has been shown to be functionally involved in wound healing and whose expression is expected to increase upon treatment [50,51,52]. MMP-2 and MMP13 have been found in acute and chronic wounds and can serve a fundamental function in controlling extracellular matrix breakdown and deposition, resulting in wound epithelialization. The expression level of FGF-1, MMP-2, and MMP-13 genes significantly up-regulated in cells treated with Nio@GelMa (1.3-, 1.1-, and 1.3-fold, respectively), Thymol (2.4-, 2.2-, and 3.3-fold, respectively), Nio–Thymol (4.6-, 3.5-, and 4.3-fold, respectively), and Nio–Thymol@GelMa (5.7-, 4.1-, and 5.1-fold, respectively) compared with those in the control treated with medium (Figure 4D).

All tested genes showed significantly higher up-regulation in cells treated with Nio–Thymol and Nio–Thymol@GelMa compared with those with Thymol treatment (*p* < 0.001), with Nio–Thymol@GelMa leading to the highest up-regulation. These results revealed the great potential of Nio–Thymol@GelMa as a candidate for wound healing over the free or niosomal-encapsulated form of Thymol. Previous studies have shown that niosomes can be involved in accelerating wound healing by prolonging drug release time [53]. In particular, when GelMa is used, the degradation product gelatin may be processed into aminophenol and subsequently utilized by the cells, avoiding the generation of toxic by-products. When it is applied to the body for wound healing, the biodegradable hydrogel formulation can promote cell proliferation and cell reorganization [50].

## 4. Conclusions

In this work, the antimicrobial and wound-healing properties of Thymol-loaded niosomes in combination with GelMa was investigated. Nio–Thymol@GelMa was found to be able to enhance biocompatibility, stability, and controlled drug release compared with free and niosomal Thymol. Consequently, Nio–Thymol@GelMa formulation was more potent in killing both Gram-positive and Gram-negative pathogens, as well as eliminating biofilms in vitro. Furthermore, Nio–Thymol@GelMa exhibited low cytotoxicity against normal cell lines and dramatically promoted cell migration. Thus, this study showcased that non-cytotoxic Nio@GelMa can be utilized to deliver active agents onto infected wounds, on the one side permitting an effective killing of bacteria and biofilm removal, and on the other side enhancing cell migration, both of which are important prerequisites in the wound healing process. While this study provides valuable insights, it does not fully replicate the complex in vivo biological conditions. Thus, it is important in the next steps to validate the results, e.g., the antibacterial efficacy and cytocompatibility through in vivo experiments. The in vitro findings obtained here can serve as a foundation for future in vivo studies.

## Figures and Tables

**Table 1:** Design of experiments using the Box–Behnken method to optimize the niosomal formulation of Thymol.

Run	Levels of Independent Variables			Dependent Variables		
	Span 60 (mM)	Cholesterol (mM)	Volume Hydration (mL)	Average Size (nm)	PDI	Entrapment Efficiency (EE) (%)
1	3	0.5	6	219	0.24	70
2	5	0.5	8	194	0.25	68
3	1	1.5	6	270	0.42	62
4	3	0.5	10	165	0.3	67
5	3	1.5	8	190	0.24	64
6	1	0.5	8	212	0.29	60
7	3	2.5	6	230	0.35	53
8	3	1.5	8	183	0.25	65
9	5	1.5	6	216	0.18	62
10	1	1.5	10	223	0.23	47
11	5	2.5	8	205	0.21	57
12	3	2.5	10	236	0.26	51
13	1	2.5	8	242	0.28	45
14	3	1.5	8	199	0.23	66
15	5	1.5	10	181	0.23	57

## Data Availability

The data presented in this study are available upon request.

## Supplementary Materials

The following supporting information can be downloaded at: https://www.mdpi.com/article/10.3390/pharmaceutics15061699/s1, Table S1. Different levels for variables in the Box–Behnken design optimization. Table S2. Primers and their sequences used in real-time PCR. Table S3. ANOVA statistical analysis for the quadratic polynomial model for size, PDI, and EE. Table S4. Predicted models of Thymol-loaded niosomes. Table S5. Summary of results of regression analysis for response size, PDI, and EE for fitting to the quadratic model. Table S6. Desirability criteria and predicted values for the variables. Table S7. The optimized responses obtained using the Box–Behnken method and the experimental data for the same responses under the optimum conditions. Table S8. The kinetic release models and the parameters obtained for optimum niosomal formulation. Figure S1. Box–Behnken method for average diameter as a function of the parameters. (A–C) Response surfaces for particle size, (D–F) for PDI, and (G–I) Entrapment Efficacy as a function of the (A–C) models. Figure S2. Stability of the optimized niosomes stored for 2 months at 4 ± 2 °C and 25 ± 2 °C, re-spectively, measured by changes in size (A,B), PDI (C,D) and EE% (E,F) of Nio-Thymol and Nio-Thymol@GelMa, respectively. * *p*-value < 0.05, ** *p*-value < 0.01, and *** *p*-value < 0.001.
