# Supplementary material for: Encapsulation of Thymol in Gelatin Methacryloyl (GelMa)-Based Nanoniosome Enables Enhanced Antibiofilm Activity and Wound Healing
Source: Pharmaceutics. 2023 Jun 9;15(6):1699. doi: 10.3390/pharmaceutics15061699 (PMC10303142; doi:10.3390/pharmaceutics15061699)
Supplement: Supplementary file 1 [file pharmaceutics-15-01699-s001.zip › pharmaceutics-2403078-supplementary.pdf]

pharmaceutics

Encapsulation of Thymol in Gelatin Methacryloyl (GelMa)-Based Nanoniosome Enables Enhanced Antibiofilm Activity and Wound Healing

Maryam Moghtaderi [1,†], Saba Bazzazan [2,†], Ghazal Sorourian [1,†], Maral Sorourian [1], Yasaman Akhavanzanjani [3], Hassan Noorbazargan [4,*] and Qun Ren [5,*]

3. Results and Discussion

3.1. Analysis variance of niosome fabrication

3.1.1. Particle size analysis

Span 60 (mM), cholesterol content (nM), and hydration volume (mL) were evaluated as individual variables (**Table S1**). In contrast, particle size, polydispersity index (PDI), and entrapment efficiency (EE) were dependent factors in the optimization studies. **Table 1** shows the results of the Box–Behnken experiments. The vesicle size of Thymol loaded in niosomal formulation (Nio–Thymol) ranged between 165.9 and 270.4 nm. The analysis of variance for particle size is shown in **Table S3**. The polynomial response was modified in the quadratic model. The model was found to be significant when the p-values were less than 0.05. It shows that the individual parameters A (Span 60), B (cholesterol content), C (hydration volume), AB, and A^2 could all have a significant impact on particle size.

Table S1. Different levels for variables in the Box–Behnken design optimization.

Level	-1	0	+1
A (Span 60 content, mM)	1	3	5
B (Cholesterol content, mM)	0.5	1.5	2.5
C (Volume hydration, mL)	6	8	10

Table S2. Primers and their sequences used in real-time PCR.

Gene	Forward Primer	Reverse Primer
FGF-1	5′- GTGGATGGGACAAGGGACAG-3′	5′- GGCAGGGGGAGAAACAA-GAT-3′
MMP-2	5′-GATCTTGACCAGAATACCATC-3′	5′-GCCAATGATCCTGTATGTG-3′
MMP-13	5′-CCTTGATGCCATTACCAGTCTCC -3′	5′-AAACAGCTCCGCATCAAC-CTGC-3′

Table S3. ANOVA statistical analysis for the quadratic polynomial model for size, PDI, and EE.

Source	*f*-Value	*p*-value prob > *f*	
	**Particle size (nm)**		
Model	7.86	0.0177	Significant
A	20.84	0.0060	
B	13.66	0.0141	
C	15.29	0.0113	
A^2	7.30	0.0427	
C	17.94	0.0082	
	**PDI**		
Model	4.81	0.0492	Significant
A	15.83	0.0105	
AC	13.60	0.0142	
	**EE (%)**		
Model	9.76	0.0109	Significant
A	11.50	0.0194	
B	49.14	0.0009	
C	8.37	0.0341	
A^2	11.95	0.0181	

As presented in **Table S4**, the particle size regression model shows the increased influence of individual variable B and the possible decreased effect of A and C variables on particle size. As indicated in **Table S5**, adjusted R-squared is thought to be the closest approximation to R squared. In this perspective, the closer the Adj R Squared value is to R-Squared, the superior the model's potential to predict reactions. Adj R-squared and R-square should be within 0.2 of each other to be in good accordance. As shown in **Figure S1-A-C**, the particle size of Nio–Thymol was raised and lowered in the different values of Span 60 with the rise in cholesterol content and hydration volume value, respectively. The smallest particle size was found when the cholesterol level was low and the hydration volume was high. This observation may support this discovery that the presence of cholesterol can increase bilayer hydrophobicity, resulting in a reduction in free energy of the surface and, as a result, a reduction in particle size [1]. It could also possibly be attributed to Span 60's reduced hydrophobicity.

Table S4. Predicted models of Thymol-loaded niosomes.

Models
**Particle size (nm)** = +190.80-18.91 * A+15.31* B-16.20 * C-4.85* A * B+3.02* A * C+14.92* B * C+16.47 * A2+ 6.67 * B2+ 15.70 * C2
**PDI** =+0.24-0.044 * A+2.625E-003* B- 0.022* C-8.000E-003* A * B +0.058* A * C -0.036* B * C-2.958E-003* A2+ 0.021 * B2+ 0.029 * C2
**EE (%)** = +65.63+ 3.66 * A-7.57* B- 3.12* C+1.12* A * B +2.63* A * C +0.30* B * C- 5.49 * A2- 2.25 * B2-2.79 * C2

Table S5. Summary of results of regression analysis for response size, PDI, and EE for fitting to the quadratic model.

Response	R-squared	Adj R-Squared	Adeq Precision	Lack of fit
**Particle size**	0.9339	0.8151	9.865	0.2772
**PDI**	0.8965	0.7102	7.962	0.0892
**EE (%)**	0.9461	0.8492	10.444	0.0719

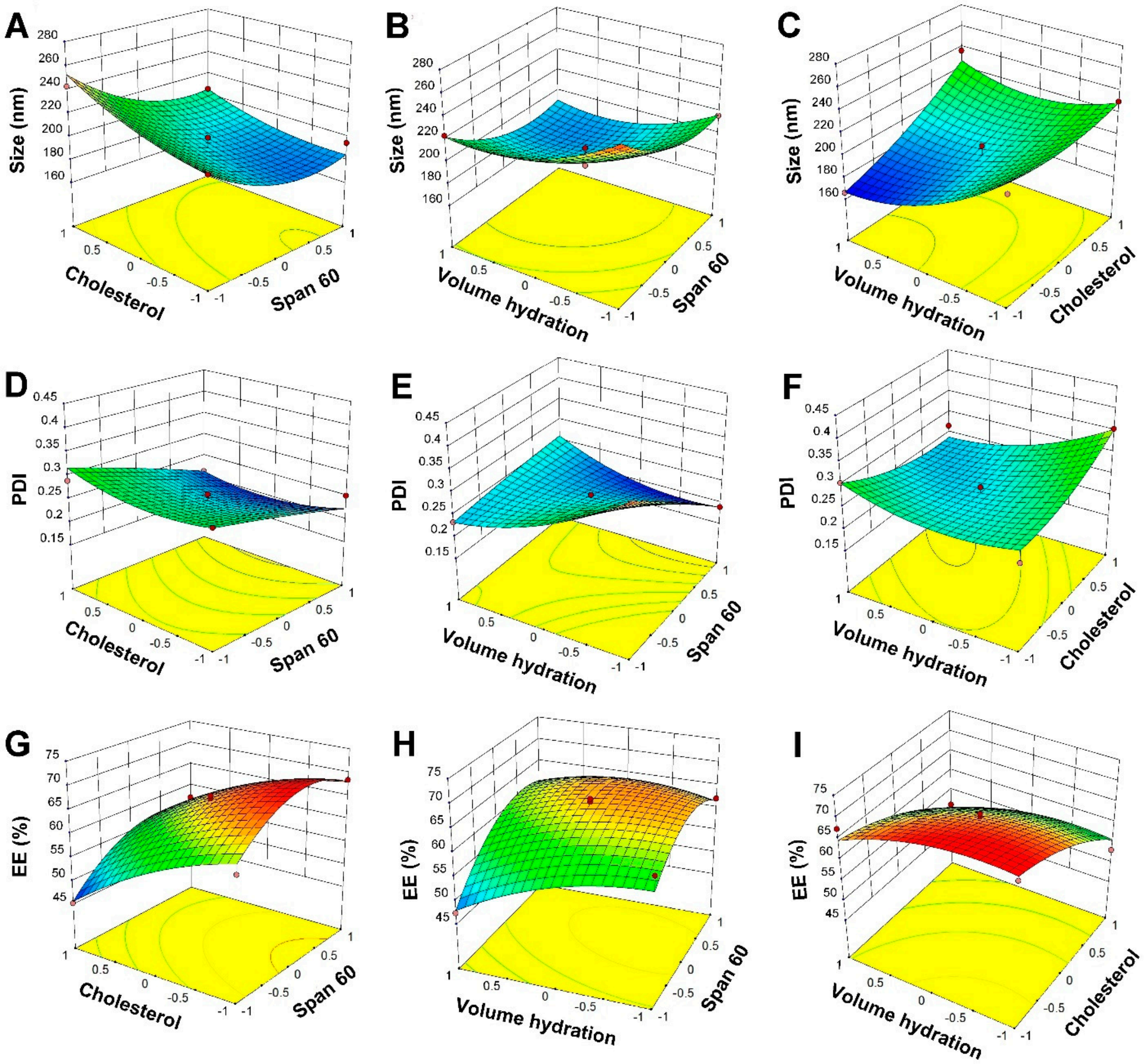

Figure S1. Box–Behnken method for average diameter as a function of the parameters. **(A-C)** Response surfaces for particle size, **(D-F)** for PDI, and **(G-I)** Entrapment Efficacy as a function of the (A, B, and C) models.

3.1.2. Polydispersity index (PDI) analysis

Table 1 presents that the PDI of Nio–Thymol ranged from 0.189 to 0.421. **Table S3** reflects the statistical analysis of PDI and reveals that PDI was substantially impacted by A, B, and AC individual variables. **Table S4** depicts the regression model for Free Thymol's PDI, which also revealed the declining effectiveness of individual variables A, B, and C on drug PDI. **Figure S1-D-F** shows the response surface plot of the PDI of Nio–Thymol. It can be concluded that PDI decreased at low span values with increasing hydration volume values, but did not show a significant effect on PDI change with increased cholesterol contents. Previous studies have revealed that the kind of surfactant seems to substantially impact the particle size and PDI of niosomes. According to the findings, the size of niosomes increased consistently as the surfactant HLB levels increased (i.e., Span 60).

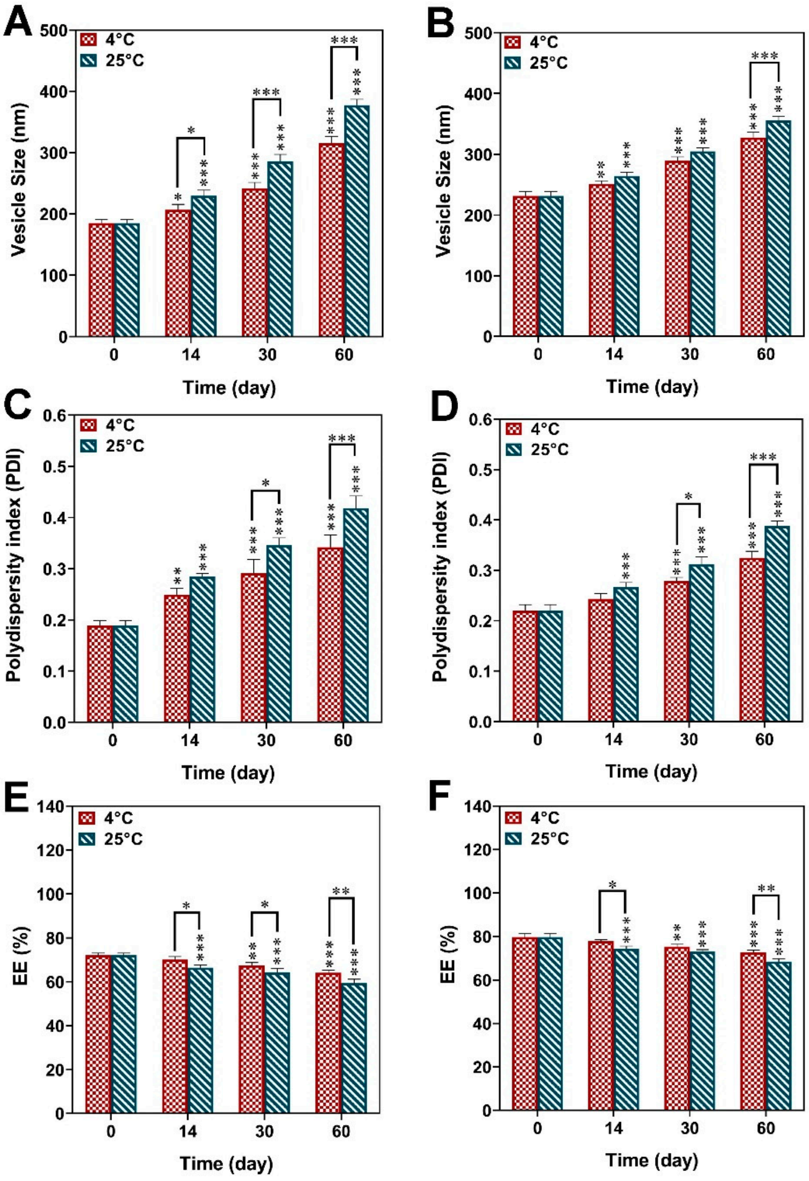

Figure S2. Stability of the optimized niosomes stored for 2 months at 4 ± 2 °C and 25 ± 2 °C, respectively, measured by changes in size **(A,B)**, PDI **(C,D)** and EE% **(E,F)** of Nio-Thymol and Nio-Thymol@GelMa, respectively. * *p*-value < 0.05, ** *p*-value < 0.01, and *** *p*-value < 0.001.

3.1.3. Entrapment efficiency analysis

Table 1 shows that Nio–Thymol's entrapment effectiveness (EE %) ranged from 45.12% to 70.58 %. **Table S3** provides the data analysis of EE percent, which demonstrated that A, B, C, and A2 individual variables had a significant impact on EE percent. **Table S4** displays the regression model for the EE %, which shows that individual variables have rising efficacy on EE%, whereas variables B and C have decreasing effects on the EE %. **Figure S1-G-I** depicts the EE % of the Nio–Thymol response surface approach. It can be inferred that the percentage of EE declined in various span values with increasing amounts of cholesterol content and hydration volume, and it was also documented that the highest percentage of EE was reported in higher span values and low amounts of cholesterol content, as well as intermediate amounts of hydration volume. This stated behavior can be attributable to two main factors: 1) Elevated cholesterol increases bilayer hydrophobicity and stability, reducing bilayer permeability, perhaps resulting in efficient hydrophobic component trapping in bilayers as the vesicle develops. 2) The increased en-

trapment might well be related to the surfactant's solid nature, hydrophobicity, and increased phase transition temperature (Span 60), and experimental results were comparable to those described in the research for carboxyfluorescein niosomes and zidovudine niosomes [2, 3]. The optimum conditions for Nio–Thymol formulation and the obtained results are listed in **Table S6, 7**.

Table S6. Desirability criteria and predicted values for the variables.

Number	Hydration volume mL	Cholesterol content mM	Span 60 content mM	Desirability
1	4.1	0.73	8.31	0.88

Table S7. The optimized responses obtained using the Box–Behnken method and the experimental data for the same responses under the optimum conditions.

Parameter	Predicted by RSM	Nio–Thymol	Nio–Thymol@GelMa	Empty niosome (Nio)
Average size (nm)	175	184±6	231±7	139±5
PDI	0.23	0.18±0.01	0.22±0.01	0.17±0.00
Entrapment Efficiency (EE) (%)	69	72±1	79±1	-
Zeta potential (mV)	-	-21±1	-10±1	-28±1

Table S8. The kinetic release models and the parameters obtained for optimum niosomal formulation. * Diffusion or release exponent.

**Release Model**	Equation	R^2				
		Free Thymol (Thy) (pH=7.4-37°C)	Nio–Thymol (pH=7.4-37°C)	Nio–Thymol (pH=6.5-37°C)	Nio–Thymol@GelMa (pH=7.4-37°C)	Nio–Thymol@GelMa (pH=6.5-37°C)
**Zero-Order**	$C_t=C_0+K_0t$	R^2=0.6552	R^2=0.7918	R^2=0.7984	R^2=0.8138	R^2=0.8051
First-Order	$\text{LogC}=\text{LogC}_0+Kt/2.303$	R^2=0.9535	R^2=0.8402	R^2=0.9030	R^2=0.8518	R^2=0.8729
Higuchi	$Q=K_H\sqrt{t}$	R^2=0.8217	R^2=0.9248	R^2=0.9285	R^2=0.9379	R^2=0.9324
Korsmeyer–Peppas	$M_t/M_\infty=K_t^n$	R^2=0.8885 n* =0.4199	R^2=0.9483 n* =0.4967	R^2=0.9529 n* =0.5060	R^2=0.9533 n* =0.5442	R^2=0.9390 n* =0.4896

3.3.2. In vitro release profile and kinetic study

As observed in **Table S8**, both the Korsmeyer–Peppas and Higuchi models were the best models explaining the kinetics of the Thymol releases from Nio–Thymol and Nio–Thymol@GelMa formulations in various PHs, where the parameter 'n' showed the drug release regime/mechanism. The release outcomes from Nio–Thymol and Nio–Thymol@GelMa formulations preceded the Korsmeyer–Peppas kinetic model with n = 0.4967 and 0.5442 at pH = 7.4 for Thymol release, respectively, (i.e., n = 0.43 and n=0.50 indicate the Fickian diffusion mechanism), while parameter n increased in acidic circumstances and attained n > 0.6 (i.e., 0.43 < n < 0.93 and 0.50<n<0.96 indicates the Anomalous transport mechanism). At acidic pH, the niosomal architecture swelled/broke down, explaining the change in the drug release mechanism. **Table S8** also demonstrates the First-Order model as the proper model representing the unreleased Free Thymol solution.
